# A novel approach to translymphatic chemotherapy targeting sentinel lymph nodes of patients with oral cancer using intra-arterial chemotherapy - preliminary study

**DOI:** 10.1186/1758-3284-3-42

**Published:** 2011-09-19

**Authors:** Junkichi Yokoyama, Shin Ito, Shinichi Ohba, Mitsuhisa Fujimaki, Katsuhisa Ikeda

**Affiliations:** 1 Department of Otolaryngology, Head and Neck Surgery, Juntendo University School of Medicine, Tokyo, Japan

## Abstract

**Background:**

Recent progress in ICG (indocyanine green) fluorescence imaging has provided a means by which we can detect sentinel lymph nodes (SLNs) without the risk of exposing patients to radiation. Neck metastasis is the most significant prognostic factor. It is imperative that early metastasis can be controlled. Intra-arterial chemotherapy is performed in order to preserve organs and to improve prognosis when treating oral cancer.

**Objective:**

Evaluate translymphatic chemotherapy targeting SLNs in patients with oral cancer using intra-arterial chemotherapy. Evaluation will be carried out through the measurement of CDDP concentrations in SLNs.

**Methods:**

Five patients with tongue cancer (T3N0M0) were treated by intra-arterial chemotherapy as neoadjuvant chemotherapy from November 2010 to June 2011. After a week of chemotherapy, patients underwent surgical treatment including the partial resection of the tongue and neck dissection. Intra-arterial chemotherapy was administered at 50 mg/m^2 ^of CDDP either one or two times weekly. 5 mg of ICG was administered to the lingual artery at the beginning of surgery. SLNs were detected using ICG fluorescence imaging. 0.1 g of SLNs and non-SLNs were resected in order to measure CDDP concentrations. The rests of each of the SLNs were examined intraoperatively by routine frozen pathological examination. SLNs were also identified using radioactive injection the day prior to surgery.

**Results:**

The mean CDDP concentrations in the SLNs and non-SLNs were 1.2 μg/g and 0.35 μg/g, respectively. Our intra- arterial infusion revealed that all metastatic lymph nodes, including SLNs, were without false negative SLNs. However, of 7 metastatic lymph nodes, one was not identified by means of conventional method.

**Conclusion:**

Our findings verified the possibility that intra-arterial chemotherapy may be effective not only for organ preservation therapy, but also efficient in translymphatic chemotherapy targeting SLNs in patients with oral cancer through the use of ICG fluorescence imaging.

## Introduction

The sentinel lymph node (SLN) is defined as the lymph node that firstly receives lymphatic drainage from the primary cancer [1]. The SLN is thought to be the first possible micrometastatic site via lymphatic drainage from the primary cancer. Thus, the pathological status of the SLN can predict the status of all regional lymph nodes. If the SLN is recognized as being negative for cancer metastasis, unnecessary dissection may be avoided and a positive prognosis achieved. This SLN concept is well established in the treatment of patients with several types of solid carcinomas, such as melanoma and breast cancer [2-4]. The SLN concept has revolutionized the approach to surgical staging of both the melanoma and breast cancer, and these techniques can benefit patients by preventing various complications due to unnecessary prophylactic dissection when the SLN is negative for cancer metastasis. Recently, the SLN concept has been extended to many other solid tumors, including head and neck cancers [5,6]. In this study, we consider a newly developed translymphatic chemotherapy procedure targeting the SLN using intra-arterial chemotherapy for oral cancer to improve prognosis and to preserve significant organs [7-9].

### Objective

Evaluate CDDP concentrations in SLNs and non-SLNs. Determine the usefulness of translymphatic chemotherapy targeting SLNs in patients with oral cancer using intra-arterial chemotherapy.

### Method and Patients

**Figure 1:**
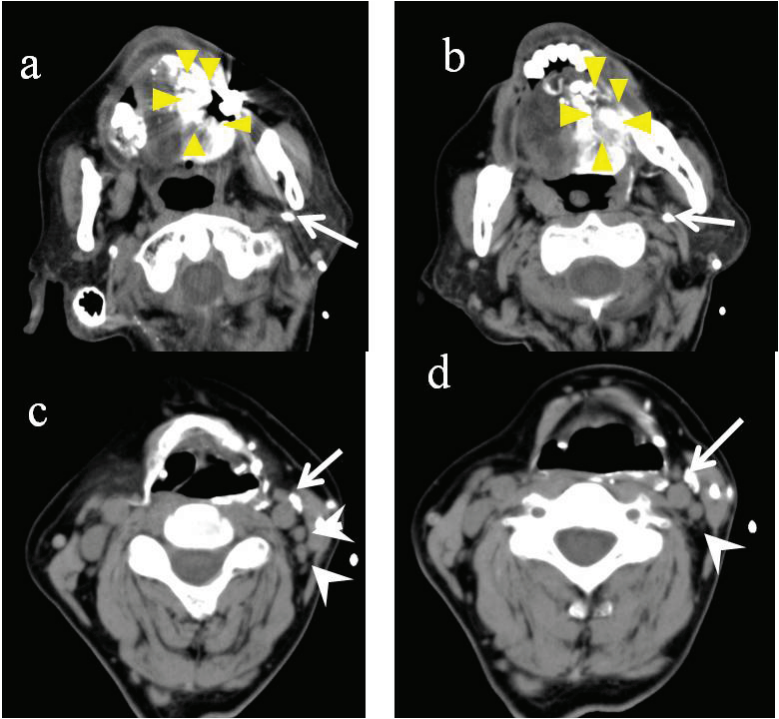
**CT-angiography infusing the lingual artery**. CT-angiography confirmed the stained tongue cancer (a and b) indicated by triangls. There was no staining in any lymph nodes (c and d) indicated by arrowheads. Arrows represent the catheter inserted in the lingual artery.

**Figure 2:**
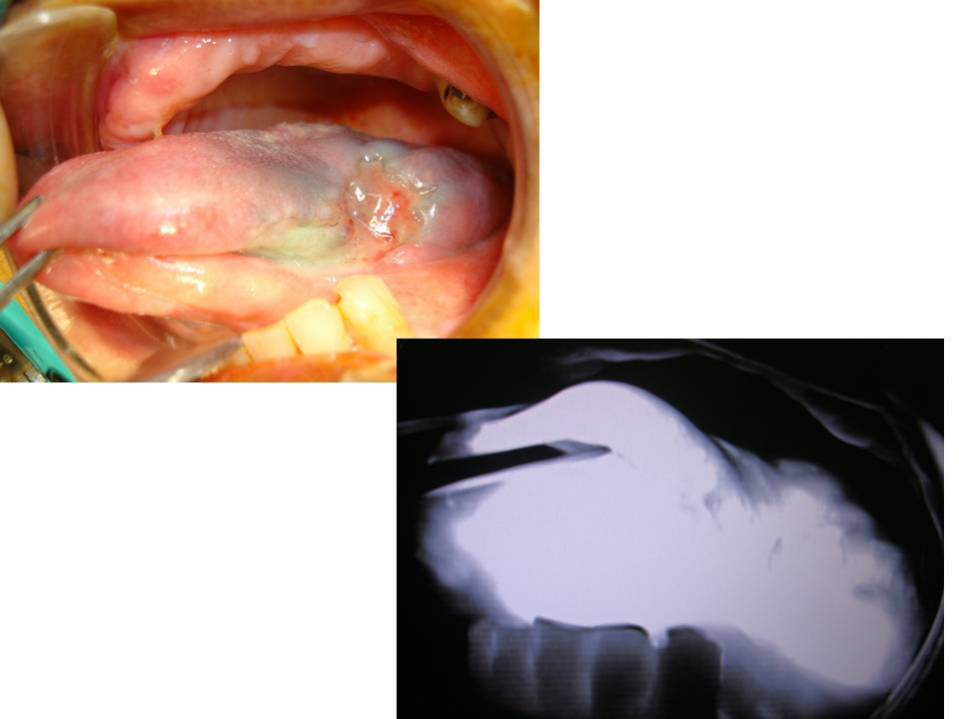
**Tongue cancer after injection of ICG**. a: tongue cancer, b:tongue cancer with ICG fluorescence imaging.

Five patients with tongue cancer (T3N0M0) were treated by intra-arterial chemotherapy as neoadjuvant chemotherapy from November 2010 to June 2011. After a week of chemotherapy, surgical treatment including partial resection of the tongue and neck dissection was performed. Intra- arterial chemotherapy was administered at 50 mg/m^2 ^of CDDP either one or two times weekly. CT-angiography confirmed that the areas of tongue cancer were stained and that lymph nodes were not stained (Figure 1). Five mg of ICG was administered via a catheter positioned in the lingual artery at the beginning of the surgery (Figure 2). SNLs were detected by ICG fluorescence imaging (Photodynamic Eye, Hamamatsu Photonics) and non-SNLs were detected in two submandibular lymph nodes located near the tongue cancer. These were monitored as controls. In order to measure CDDP concentrations, 0.1 g of each of the SLNs and the two non-SLNs were resected and the rests of each of the SLNs were examined intraoperatively by means of routine frozen pathological examination. The CDDP concentrations were measured by atomic absorption analysis. A conventional method of identifying SLNs using radioactive injection was also performed the day before surgery. The pre-treatment characteristics of the patients are shown in table 1.

**Table 1:** Patients characteristics

cases	site	age	M/F	TNM	No of SLNs by radiocolloid	No of SLNs by ICG	No of non-SLNs
**1**	tongue	34	M	T3N0M0	3	4	11

**2**	tongue	57	F	T3N0M0	3(FN)	6	21

**3**	tongue	37	M	T3N0M0	3	6	14

**4**	tongue	63	M	T3N0M0	4	6	16

**5**	tongue	59	M	T3N0M0	4	7	28

Mean		50			3.4	5.6	18

FN: False Negative, LN: Lymph node

Patients' informed consent was obtained prior to treatment, and this study was approved by the Human Ethics Review Committee of Juntendo University.

The difference between the two groups CDDP concentrations were tested by Student's t- test and Wilcoxon test.; p values < 0.05 were considered to indicate significance.

## Results

**Figure 3:**
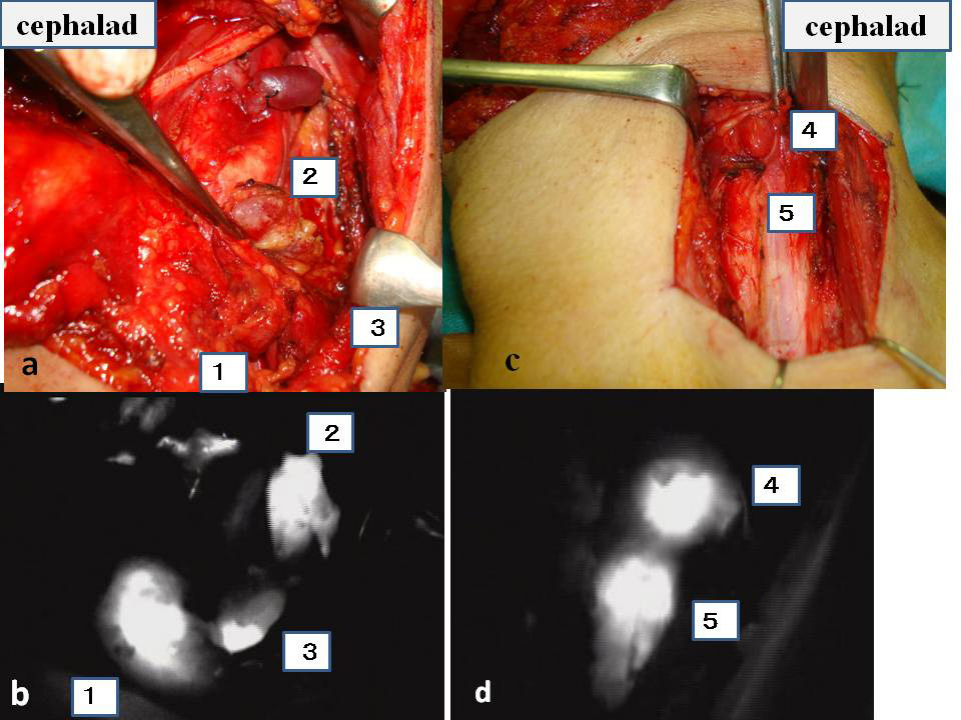
**Intraoperative navigation surgery using ICG fluorescence imaging**. Number(1~5) means SLNs. a and b represent level II and III dissection. c and d represent level III and IV dissection.

**Figure 4:**
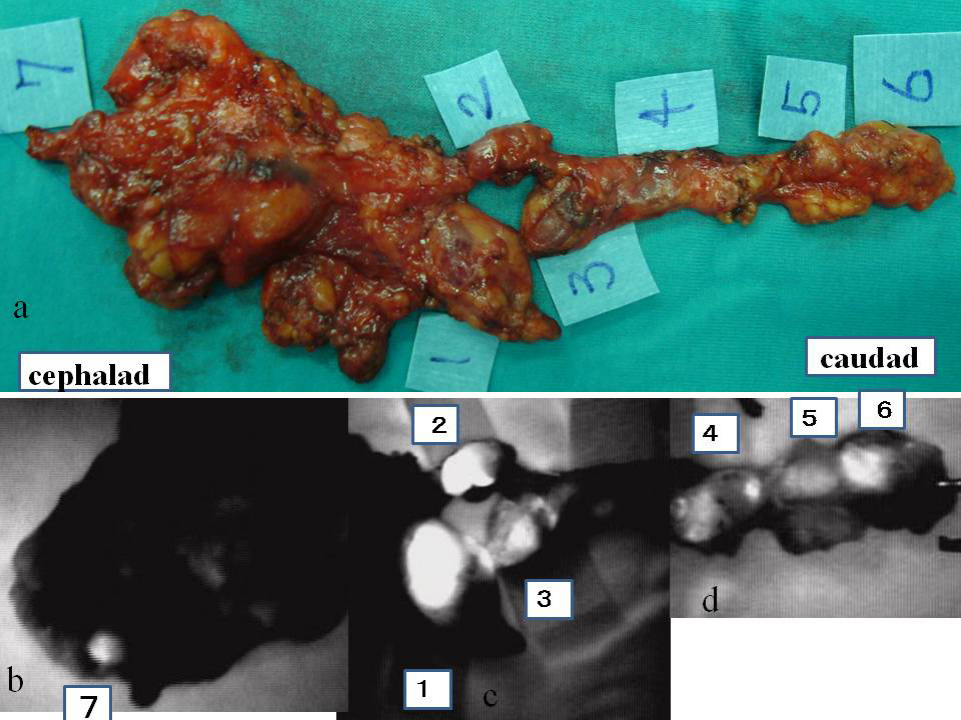
**Resected specimens**. a:Rt side represents the caudal side. Number(1~7) represents SLNs. b:level I, c: level II and III, d: level III and IV.

Detection of SLNs were clearly demonstrated by ICG fluorescence imaging (Figure 3, 4). The mean number of SLNs was 5.6 (3-8). ICG fluorescence imaging showed a greater number of SLNs in our intra-arterial infusion than seen when injecting radiocolloid intratumor (mean 3.4). SLNs detected by ICG fluorescence imaging included all of the SLNs detected by the conventional radioactive method.

Histopathological examination was performed for 29 SLNs and 90 non-SLNs (Table 1). All 5 patients with histopathologically verified metastasis in their SLNs demonstrated positive results in ICG fluorescence imaging. No false negative cases were identified within each SLN basin. However, of the 7 metastatic lymph nodes, one was not identified by means of conventional methods.

The mean CDDP concentrations of SLNs and non-SNLs were 1.2 μg/g and 0.35 μg/g respectively. The CDDP concentration of SLNs was significantly higher than non-SLNs. The mean CDDP concentration of tongue cancer was 2.3 μg/g.

No hematological complications were caused by intra-arterial chemotherapy. All patients are alive with no evidence of disease and are able to consume food as they were able to before surgery.

## Discussion

Chemoradiation therapy has significantly enhanced the preservation of important organs in the treatment of head and neck cancer. However, because of severe mucositis and low sensitivity to chemotherapy, tongue cancer has not been treated by chemoradiation as often as other sites of head and neck cancer [10]. CDDP is a most promising drug for the treatment of head and neck cancers. To increase the CDDP concentration in tongue cancer resistant to chemotherapy we have adopted intra-arterial chemotherapy for the treatment of advanced tongue cancer. This procedure has resulted in a positive prognosis and good organ preservation [7,9]. We found that the administration of CDDP to the primary tongue cancer has a powerful effect on the primary cancer as well as occult neck metastasis.

**Figure 5:**
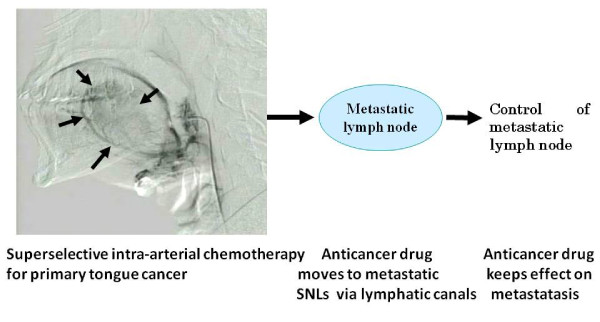
**The schema of translymphatic chemotherapy using intra-arterial chemotherapy**. CDDP administered to the primary tongue cancer moves selectively to SLNs via lymphatic canals and CDDP accumulated in SLNs. The arrows indicate cancer.

As a result, we have hypothesized that intra-arterial chemotherapy for the treatment of primary tongue cancer, also results in translymphatic chemotherapy to control the subclinical metastatic tumor in SLNs. The schema of translymphatic chemotherapy is illustrated in Figure 5. This schema shows that CDDP administered to the primary tongue cancer moves selectively to SLNs via lymphatic canals. CDDP is accumulated in the SNLs and results in a high CDDP concentration in the SLNs. Compared with the 2.3 μg/g CDDP concentration measured within the tongue cancer, the mean CDDP concentration measured in SLNs was recorded at 1.2 μg/g. However, the difference between the CDDP concentrations of SLNs and tongue cancer was significant.

**Figure 6:**
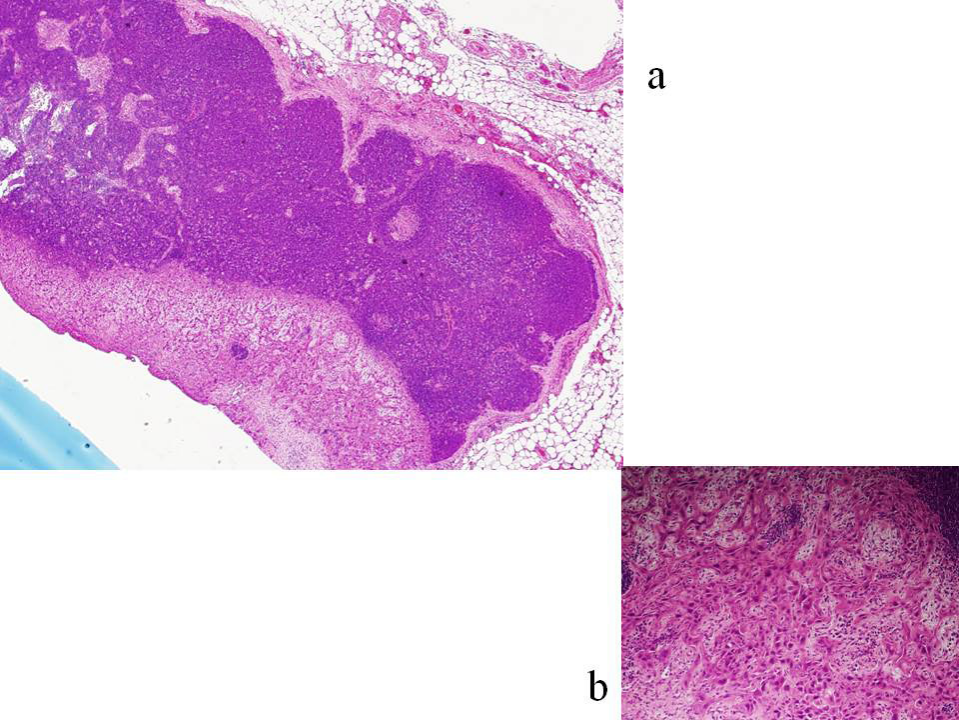
**A metastatic SLN not detected by the conventional method**. a: left side low power magnification. b: right side high power magnification. This lymph node contained CDDP as high as 1.68 μg/g.

In our preliminary study, all SLNs were detected by ICG fluorescence imaging infused via the lingual artery in 5 cT3N0 tongue cancer patients. The number of SLNs resulting from intra-arterial infusion was greater than could be seen when by means of conventional injection to the intratumor. This is because ICG was administered to the lingual artery and ICG spread throughout half of the tongue (Figure 2). ICG moved via lymphatic canals from half of the tongue including the tongue cancer. Even in micrometastatic SLNs, an afferent lymphatic sometimes occluded by micrometastatic cancer based on sentinel navigation or CT lymphograpy [11]. In our examination, we also did not detect a metastatic SLN by conventional methods due to occlusion of afferent lymphatics from the tongue cancer (Figure 6). It contained CDDP as high as 1.68 μg/g. This was because, each lymph node has several afferent lymphatics and ICG or CDDP could move to micrometastatic SLN via several other afferent lymphatics in the case of intra- arterial infusion. CDDP was released continuously from the primary tongue cancer via the translymphatic canal for a period of over more than one week. CDDP was selectively accumulated in SLNs and continued to effect micrometastasis in SLNs over a long period. After a period of several weeks, the CDDP concentrations between the primary cancer and SLNs gradually will become the same and maintained equilibrium. Our intra- arterial chemotherapy is suspected to contribute not only to primary organ preservation, but also to a positive prognosis by controlling the metastatic SLNs. Preservation of patients quality of life in advanced cT3N0 tongue cancer is achieved by means of intra- arterial chemotherapy and through targeting SLN metastasis with translymphatic chemotherapy. We believe that ICG fluorescence imaging is very useful for navigation surgery as there appear to be no limitations.

An additional reason for difficulties in detecting SLNs was the close proximity of the primary tumor to the lymph node basin. This caused difficulties for both preoperative lymphoscintigraphy and intraoperative radiolocalization, because of the well described phenomena of "shine-through'' radioactivity and scatter from the primary site [4]. Specifically, it was particularly difficult to detect SLNs on the floor of the mouth in any other sites of head and neck cancers [12,13]. In order to avoid the influence of 'shine-through' we firstly resected the close primary tumor before sentinel mapping. However, it was difficult to completely avoid the influence of 'shine-through' after resection of the primary tumor. As for ICG fluorescence imaging, SLNs were clearly detected even in close proximity to the primary tumor and 'shine-through' could be avoided. The ICG fluorescence imaging procedure demonstrated better success rates of detecting SLNs for patients with tumors in the floor of the mouth than the radioactivity method.

Further studies will be required to verify the effectiveness and safety of intra-arterial chemotherapy as a method of lymphatic chemotherapy for the treatment of occult lymph node metastatsis. Our results suggest that a drug delivery system based on the SLN concept should be developed for local chemotherapy targeting SLNs in patients with cN0 oral cancer, for whom there is potential for metastasis in SLNs.

Further investigations may lead to the development of a new minimally invasive multimodal therapy targeting both the primary tumor and SLNs in the near future.

## Conclusion

Our study verified the possibility that intra-arterial chemotherapy may be effective not only for organ preservation therapy, but also serve as an efficient procedure for translymphatic chemotherapy targeting SLNs in patients with oral cancer through the use of ICG fluorescence imaging. The CDDP concentrations recorded in SLNs were significantly higher than in non-SNLs.

This novel drug delivery system is feasible for translymphatic chemotherapy targeting SLNs in patients with cT3N0 oral cancer with the possibility of occult metastasis in SLNs.

## Competing interests

The authors declare that they have no competing interests.

## Authors' contributions

JY and SI prepared and edited this manuscript. SO and MF contributed to the collection of data.

KI performed the statistical analysis. JY and KI gave final approval for this version of the manuscript. All authors read and approved the final manuscript
